# 非小细胞肺癌患者凝血功能异常的相关因素与预后分析

**DOI:** 10.3779/j.issn.1009-3419.2014.11.04

**Published:** 2014-11-20

**Authors:** 艳华 李, 素菊 魏, 俊艳 王, 雷 洪, 利格 崔, 彩 王

**Affiliations:** 050000 石家庄，河北医科大学第四医院肿瘤内科 Department of Oncology, the Fourth Hospital of Hebei Medical University, Shijiazhuang 050000, China

**Keywords:** 肺肿瘤, 凝血功能, 高血凝状态, 预后, Lung neoplasms, Blood coagulation, Thrombophilia, Prognosis

## Abstract

**背景与目的:**

凝血及纤溶系统激活在肺癌患者中较为常见，与肺癌侵袭、转移的高风险和预后差相关。非小细胞肺癌（non-small cell lung cancer, NSCLC）占肺癌的80%-85%，本研究旨在回顾性分析凝血功能指标对NSCLC的预后价值，为目前NSCLC患者高凝状态的预防及治疗提供参考。

**方法:**

回顾性分析2009年1月-2012年12月首次就诊于河北医科大学第四医院的604例经病理学证实的NSCLC患者的临床资料。资料内容包括患者治疗前凝血功能相关指标[血浆凝血酶原时间（prothrombin time, PT）、凝血酶原活动度（prothrombin time activity, PTA）、国际标准化比率（international normalized ratio, INR）、活化部分凝血活酶时间（activated partial thromboplastin time, APTT）、纤维蛋白原（fibrinogen, Fib）、D二聚体（D-dimer, D-D）、血小板计数（platelet count, PLT）]、性别、年龄、病理分型、TNM分期、淋巴结状态等。本研究选择了50例同期就诊于河北医科大学第四医院的非癌症患者作为对照组。采用SPSS 13.0统计软件进行分析。

**结果:**

NSCLC组与对照组之间所有的凝血功能指标（包括PT、PTA、INR、APTT、Fib、D-D、血小板计数）的血浆水平显示均有统计学差异[除了Fib（*P*=0.001, 5）、Plt（*P*=0.004, 5），其余指标（*P*＜0.001）]。纤维蛋白原水平与NSCLC的组织学亚型之间相关，鳞癌比腺癌的Fib水平明显升高（*P*＜0.001）。Ⅲ期、Ⅳ期期比Ⅰ期-Ⅱ期患者的Fib、PLT水平升高（*P*＜0.001, *P*=0.014），APTT缩短（*P*＜0.001）。与N0患者相比，N1-N3患者的APTT，明显缩短（*P*＜0.001），Fib、D-D水平升高（*P*＜0.001, *P*=0.048）。对生存率的比较研究显示，PT、INR延长（*P*=0.032, *P*=0.001），Fib升高（*P*＜0.001），PTA下降（*P*=0.005），在统计学上对总生存有明显的不利影响。多因素生存分析显示，在凝血功能指标中INR是唯一的独立预后因素（*P*=0.017）。

**结论:**

NSCLC患者往往存在凝血纤溶系统的激活，导致凝血纤溶指标的亚临床改变。肺腺癌患者以及分期为晚期、淋巴结存在转移的NSCLC患者更易出现高血凝状态。PT、INR的延长与NSCLC患者生存率的下降密切相关，INR是NSCLC的独立预后因素，PT、INR可能成为NSCLC的预后指标。

## Abstract (en)

**Background and objective:**

The activation of coagulation and fibrinolysis is frequently encountered among cancer patients. Such tumors are associated with high risk of invasion, metastases, and negative final outcomes. Non-small cell lung cancer (NSCLC) accounts for approximately 80% to 85% of all lung malignancies. This study aims to investigate the prognostic value of blood coagulation tests for NSCLC and provide a reference to patients on the prevention and treatment of thrombophilia.

**Methods:**

Data were collected from 604 cases of hospitalized patients with histologically confirmed NSCLC from January 2009 to December 2012 at the Fourth Hospital of Hebei Medical University. Data included the related indexes of coagulation function in patients before treatment [(*i*.*e*., prothrombin time (PT), prothrombin time activity (PTA), international normalized ratio (INR), activated partial thromboplastin time (APTT), fibrinogen (Fib), D-dimer, and platelet count)], as well as sex, age, pathological type, TNM stage, and lymph node status. Fifty control subjects without cancer were included in the analysis. Statistical analysis was conducted by using SPSS 13.0 software.

**Results:**

The plasma level of all coagulation tests including D-dimer, Fib, PT, APTT, INR, and platelet counts revealed statistically significant differences between the patient and control group (*P* < 0.001 for all variables; *P*=0.001, 5 and *P*=0.004, 5 for Fib and platelet counts, respectively). The squamous subtype exhibited high plasma Fib levels (*P* < 0.001) compared with adenocarcinoma cell lung cancer patients. Fib and PLT levels increased (*P* < 0.001 and *P*=0.014, respectively), and aPTT decreased (*P* < 0.001) in patients at stages Ⅲ and Ⅳ compared with those in patients at stages Ⅰ and Ⅱ. aPTT decreased significantly (*P* < 0.001), and Fib and D-dimer levels increased (*P* < 0.001 and *P*=0.048, respectively) in N1-3 patients with NSCLC compared with those of N0 patients. Prolonged PT and INR, high plasma Fib levels, and low PTA levels had statistically significant adverse effects on survival (*P*=0.032, *P*=0.001, *P* < 0.001, and *P*=0.005, respectively). Multivariate analyses revealed that only increased INR was associated with a decreased survival rate in the related indexes of coagulation function (*P*=0.017).

**Conclusion:**

Patients who have lung adenocarcinoma and/or lymph node metastasis at advanced stages of NSCLC are prone to thrombophilia. Prolonged PT and INR was associated with poor prognosis, and elevated INR was independently associated with a decreased survival rate. PT and INR are promising prognostic markers of NSCLC.

近来研究越来越多地发现凝血功能紊乱通常是恶性肿瘤的首发迹象。Kalweit等^[1]^通过对供应肺肿瘤的肺静脉血和肺癌患者外周血进行凝血纤溶指标检测，发现肺静脉血中各项指标明显高于外周静脉血，说明肺癌可直接激活凝血及纤溶系统。虽然临床上血栓性疾病和弥散性血管性凝血（disseminated intravascular coagulation, DIC）仅发生于较少的癌症患者，但是近些年的研究证实在肺癌患者中亚临床的凝血纤溶指标的改变尤为常见。因此，高血凝状态越来越受到临床医师的重视，成为近期研究的热点问题之一。

癌症和凝血系统之间存在多个机制，这些机制证明了肿瘤生物学与凝血功能是紧密相连的过程^[2]^。最近的研究表明，癌症患者的凝血异常可能导致了炎性细胞的募集、肿瘤间质的产生和血管生成。肿瘤激活凝血系统增加了其侵袭与转移的风险。研究^[3, 4]^表明高水平的循环生物标志物，如活化凝血和纤溶系统的纤维蛋白原、纤维蛋白裂解产物和D二聚体（D-dimer, D-D）等与多种类型肿瘤生存率的下降密切相关。针对肺癌患者活化的凝血系统与预后之间关系的研究已经揭示了相似的结果^[5]^。

肺癌是最常见的严重威胁人类健康的恶性肿瘤之一，非小细胞肺癌（Non-small cell lung cancer, NSCLC）占肺癌的80%-85%，本研究回顾性分析604例NSCLC患者凝血功能异常的相关临床资料，探讨凝血功能指标的预后价值，为目前NSCLC患者高凝状态的预防及治疗提供参考。

## 1 资料与方法

### 1.1 资料来源

本研究收集2009年1月-2012年12月首次就诊于河北医科大学第四医院的604例经病理学证实的NSCLC患者的临床资料。资料内容包括患者治疗前凝血功能相关指标包括血浆凝血酶原时间（prothrombin time, PT）、凝血酶原活动度（prothrombin time activity, PTA）、国际标准化比率（international normalized ratio, INR）、活化部分凝血活酶时间（activated partial thromboplastin time, APTT）、纤维蛋白原（fibrinogen, Fib）、D-D、血小板计数（platelet count, PLT）、性别、年龄、病理分型、TNM分期、淋巴结状态等。所有患者在就诊前6个月内无抗癌治疗史。肺癌的病理学诊断根据世界卫生组织（World Health Organization, WHO）标准，分期根据修订后的TNM肺癌分期[国际抗癌联盟（Union for International Cancer Control, UICC）2010分期标准]。本研究选择了50例同期就诊于河北医科大学第四医院的非癌症患者作为对照组。

### 1.2 检测方法和观察指标

采集治疗前清晨空腹静脉血，所用仪器为ACL TOP700凝血分析仪，PT、TT、APTT、Fib质控血浆N.P均为美国独立实验室产品。PT正常值9 s-12 s；APTT正常值28 s-41 s；Fib正常值2.00 g/L-4.40 g/L，PTA正常值80%-160%；INR正常值0.8-1.4。D-D检测采用干式免疫散射色谱法。所用仪器为NycoCard Reader Ⅱ检测试剂盒，D-D正常值0-1.00 mg/L。采用雅培全自动血细胞分析仪及配套试剂检测PLT正常值100×10^9^/L-300×10^9^/L。

### 1.3 随访

随访方式主要为电话、门诊及住院等，所有患者随访至2013年12月31日，随访时间最短为2个月，最长为60个月，中位随访时间为21个月。总生存期（overall surivival, OS）是指从病理确诊时间至患者死亡时间。随访期间死于其他原因、失访或研究截止时仍生存者，均记为截尾值。

### 1.4 统计学方法

应用SPSS 13.0软件进行统计分析。不符合正态分布的计量资料采用中位数描述，组间比较采用非参数检验（*Mann*-*Whitney U*检验）；用*Kaplan*-*Meier*法绘制生存曲线；生存率比较采用*Log*-*rank*检验；多因素生存分析采用*Cox*比例风险回归模型进行分析。*P*＜0.05为差异有统计学意义。

## 2 结果

### 2.1 一般情况

本研究包括604例NSCLC患者。其中385例死亡（64%），58例失访（9.6%）。其中男性409例（68%），女性195例（32%）；中位年龄60（22-86）岁，其中≥65岁的患者187例（31%），＜65岁的患者417例（69%）。腺癌370例，鳞癌234例；Ⅰ期-Ⅲa期250例，Ⅲb期-Ⅳ期354例。Ⅰ期86例，Ⅱ期59例，Ⅲa期105例，Ⅲb期82例，Ⅳ期272例；N0者213例，N1-N3者391例。

### 2.2 NSCLC患者凝血指标与对照组比较

NSCLC患者与对照组之间所有的凝血功能指标（包括PT、PTA、INR、APTT、Fib、D-D、PLT）的血浆水平显示均有统计学差异[除了Plt（*P*=0.004, 5）、Fib（*P*=0.001, 5），其余指标*P*＜0.001）]（表 1）。NSCLC患者组较对照组PT、INR明显延长，APTT缩短，Fib、D-D、Plt水平明显升高。

**1:** NSCLC肺癌患者与对照组之间凝血功能指标的比较 The values of serum coagulation tests in patients with NSCLC and controls

Coagulation tests	Patients (*n*=604)			Controls (*n*=50)		*P*
	Median	Range		Median	Range	
PT (s)	11.3	9.5-26.6		10.1	8.8-10.6	＜0.001
PTA (%)	99	33-142		114	106-139	0.001, 5
INR	1.04	0.88-2.10		0.93	0.84-0.97	＜0.001
APTT (s)	32.0	20.9-55.4		32.4	29.8-33.4	＜0.001
Fib (g/L)	4.21	2.13-10.10		2.26	1.97-3.82	＜0.001
D-dimer (mg/L)	0.2	0.1-8.2		0.1	0.1-0.2	＜0.001
PLT (×10^9^/L)	271	130-675		217	119-247	0.004, 5
NSCLC: non-small cell lung cancer; PT: prothrombin time; PTA: prothrombin time activity; INR: international normalized ratio; APTT: activated partial thromboplastin time; Fib: fibrinogen; PLT: platelet count.						

### 2.3 NSCLC患者凝血指标与其临床病理特征的关系（表 2）

**2:** NSCLC患者凝血指标与其临床病理特征的关系 Results (median) of comparisons between the coagulation tests and various clinical and pathological characteristic

Parameters	Coagulation tests						
	PT (s)	PTA (%)	INR	APTT (s)	FIB (g/L)	D-dimer (mg/L)	PLT(×10^9^/L)
Gender							
Male	11.4	98	1.04	32.5	4.27	0.2	260
Female	11.0	99	1.02	31.1	3.93	0.3	287
*P*	0.007	0.002, 5	0.013, 5	＜0.001	＜0.001	0.499	0.816
Age (yr)							
＜65	11.1	99	1.03	32.3	4.21	0.2	282
≥65	11.5	98	1.06	31.9	4.22	0.2	251
*P*	0.183	0.197	0.178	0.392	0.066	0.420	0.099
Histological type							
Adenocarcinoma	11.8	93	1.08	31.5	4.11	0.2	279
Squamous cell carcinoma	11.0	101	1.01	32.7	4.70	0.3	258
*P*	0.006, 5	0.002, 5	0.008, 5	0.176	＜0.001	0.281	0.032
TNM stage							
Ⅰ+Ⅱ	11.4	101	1.05	38.3	3.98	0.2	270
Ⅲ+Ⅳ	11.2	98	1.04	31.5	4.22	0.2	286
*P*	0.349	0.358	0.103	＜0.001	＜0.001	0.474	0.014
Lymphonode metastasis							
N0	11.0	101	1.02	32.7	3.85	0.1	258
N1-N3	11.4	96	1.04	31.8	4.27	0.3	277
*P*	0.124	0.065	0.295	＜0.001	＜0.001	0.048	0.08

#### 2.3.1 凝血功能与NSCLC患者性别之间的关系

男性较女性患者相比PT、APTT和INR延长（*P*=0.007，*P*＜0.001和*P*=0.013, 5），PTA下降（*P*=0.002, 5），Fib升高（*P*＜0.001），这表明与女性患者相比，男性患者有更高的活化凝血级联反应的趋势。不同性别组的D-D与PLT水平差异无统计学意义（*P*＞0.05）。

#### 2.3.2 凝血功能与NSCLC患者年龄之间的关系

不同年龄组的凝血功能指标差异无统计学意义（*P*＞0.05）。

#### 2.3.3 凝血功能与NSCLC患者组织学分型之间的关系

NSCLC患者中鳞癌比腺癌的Fib水平显著升高（*P*＜0.001），PT、INR下降（*P*=0.006, 5; *P*=0.008, 5），PTA升高（*P*=0.002, 5）。不同组织学分型组的APTT、D-D差异无统计学意义（*P*＞0.05）。

#### 2.3.4 凝血功能与NSCLC患者TNM分期之间的关系

NSCLC患者中Ⅲ期、Ⅳ期比Ⅰ期-Ⅱ期的Fib、PLT升高（*P*＜0.001, *P*=0.014），APTT明显缩短（*P*＜0.001）。不同分期组的PT、PTA、INR、D-D则差异无统计学意义（*P*＞0.05）。

#### 2.3.5 凝血功能与NSCLC患者淋巴结转移状态之间的关系

N1-N3的患者与N0患者相比，APTT明显缩短（*P*＜0.001），Fib、D-D水平升高（*P*＜0.001; *P*=0.048）；而同时PT、INR延长，但无统计学意义（*P*=0.124; *P*=0.295）。不同淋巴结状态组的PTA、PLT差异无统计学意义（*P*＞0.05）。

### 2.4 NSCLC患者的生存分析

#### 2.4.1 生存率的比较（表 3）

**3:** 单因素生存分析 Univariate survival analysis results for coagulation tests and clinical and pathological characteristic

Characteristic		Estimate (Median）	95%CI		Chi-Square	*P*
			Lower	Upper		
PT	＜Median	27	19.112	34.888	4.596	0.032
	≥Median	19	15.915	22.085		
PTA	＜Median	19	15.678	22.322	7.825	0.005
	≥Median	22	14.159	29.841		
INR	＜Median	27	19.586	34.414	11.399	0.001
	≥Median	16	13.022	18.978		
APTT	＜Median	22	14.897	29.103	1.639	0.2
	≥Median	20	16.755	23.245		
Fib	＜Median	34	27.936	40.064	29.761	＜0.001
	≥Median	14	10.862	17.138		
D-dimer	＜Median	18	13.121	22.897	2.628	0.105
	≥Median	14	8.044	19.956		
PLT	＜Median	22	16.259	27.741	2.014	0.156
	≥Median	18	14.701	21.299		
Gender	Male	19	16.114	21.886	1.459	0.227
	Female	28	20.009	35.991		
Age (yr)	＜65	22	17.436	26.564	2.502	0.114
	≥65	18	13.919	22.081		
Histology type	Adenocarcinoma	17	13.534	20.466	2.086	0.149
	Squamous cell carcinoma	22	15.663	28.337		
TNM stage	Ⅰ+Ⅱ	41	30.025	40.994	101.7	＜0.001
	Ⅲ+Ⅳ	15	12.864	17.136		
Lymphonode metastasis	N0	48	37.970	58.030	81.14	＜0.001
	N1-N3	14	11.458	16.542		

本研究604例患者的中位生存期为17月。通过*Log*-*rank*生存比较研究表明，PT、INR延长（*P*=0.032; *P*=0.001），PTA下降（*P*=0.005），Fib明显升高（*P*＜0.001），在统计学上对总生存有不利影响（图 1-图 3）。尽管APTT和D-D水平高于中位数的患者与低于中位数的患者相比，生存率有下降的趋势，但是差异无统计学意义（*P*=0.20; *P*=0.105）。不同的年龄、性别及组织学分型则对生存的影响无明显统计学差异（*P*＞0.05）。

**1:**
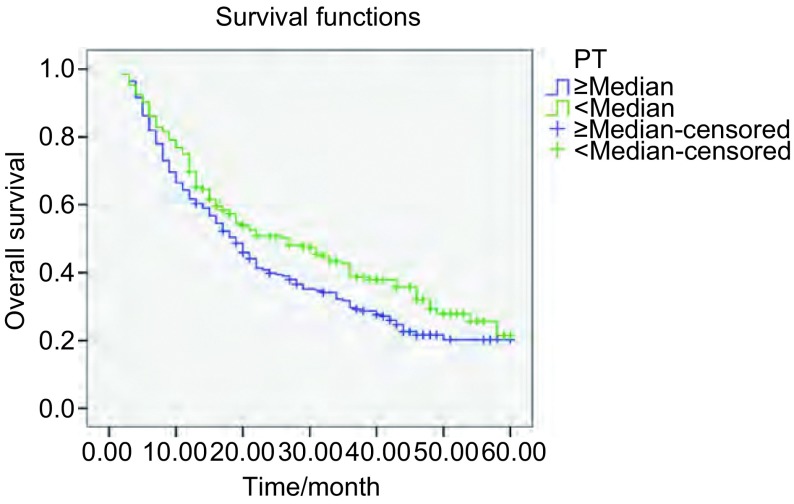
不同PT水平NSCLC患者的总生存曲线 Overall survival curves in patients with NSCLC according to PT levels (*P*=0.032)

**2:**
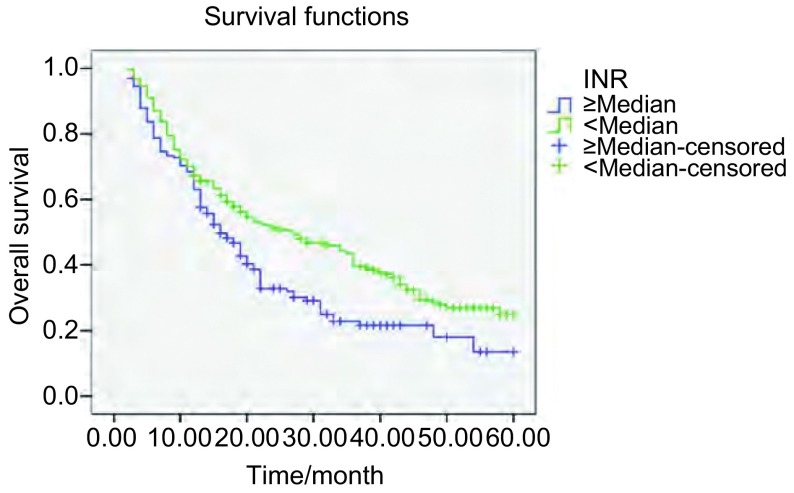
不同INR水平NSCLC患者的总生存曲线 Overall survival curves in patients with NSCLC according to INR levels (*P*=0.001)

**3:**
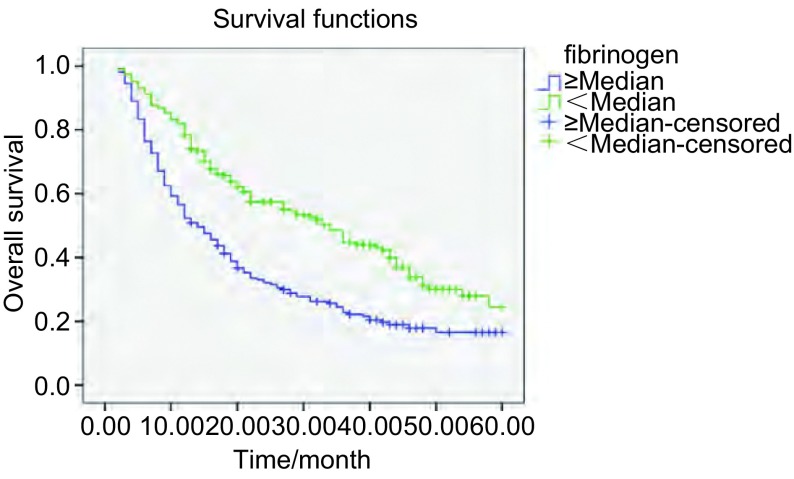
不同纤维蛋白原水平NSCLC患者的总生存曲线 Overall survival curves in patients with NSCLC according to fibrinogen levels (*P* < 0.001)

#### 2.4.2 多因素生存分析

多因素生存分析采用*Cox*比例风险回归分析（表 4）。*P*≤0.05的主要变量（包括TNM分期、淋巴结状态、INR、PTA和Fib）被纳入了分析；PT被排除是由于PT与INR的高度相关性。因此，除了TNM分期，只有延长的INR与较差的预后独立相关（TNM分期：HR=6.942，*P*=0.007；INR：HR=1.666，*P*=0.017）。

**4:** 多因素生存分析 Multivariate *Cox*'s proportional hazards model analysis

Characteristic	B	SE	Ward	df	Sig	Exp(B)
INR	0.510	0.214	5.695	1	0.017	1.666
TNM stage	1.938	0.722	7.193	1	0.007	6.942

## 3 讨论

现在已经认识到癌症患者中存在凝血系统的激活，这通常反映了常规凝血指标的亚临床异常改变。尽管许多研究讨论了恶性肿瘤患者血液高凝状态和血栓栓塞并发症的原因，但是机制仍然没有完全阐明。一些研究证明肿瘤细胞激活凝血和纤溶系统，有助于侵袭和转移。因此，在一些恶性肿瘤中（例如乳腺癌、结肠癌、肺癌），这种激活的程度与肿瘤的分期及预后密切相关^[6]^。在肺癌患者中，存在着持续的凝血刺激。肺癌患者存在凝血功能紊乱，这不仅与肿瘤的生长、浸润、侵袭、转移以及并发静脉血栓栓塞症（venous thromboembolism, VTE）等密切相关，还直接影响肺癌患者的预后。

无论是由于癌症本身还是治疗措施所致，癌症中经常存在凝血激活，典型的表现如VTE和低水平的DIC。Armand Trousseau教授于1865年首先报道了恶性肿瘤与血栓形成的相关性，肿瘤患者体内往往呈高凝状态，有并发血栓性疾病的潜在风险。迄今已有大量文献^[7]^报道恶性肿瘤患者存在凝血系统和纤溶系统功能的异常，这种异常不仅导致患者的血栓或出血症状，还与肿瘤生长、浸润、转移及血管新生等过程密切相关，而且与恶性肿瘤的预后有关。

高血凝状态是指血液发生易于形成血栓的病理生理的变化。具体表现包括：①凝血因子及纤维蛋白原合成增多或功能亢进；②纤溶及抗凝系统各蛋白或因子的合成减少、消耗增多或功能减弱；③血小板增多或功能亢进；④血管壁的损伤，启动血管内皮细胞；⑤血液粘滞度较前增高。在这种病理状态下，有助于血栓形成，若在这个时期根据某些指标的改变，早期明确血栓前状态，采取适当的措施，可能有助于改善疾病的预后。

本研究也证实了在非癌症患者对照组与NSCLC患者组中凝血功能指标水平存在明显的差别（*P*＜0.01）。在本研究中，由于凝血和纤溶系统的激活，患者组与对照组相比D-D值升高（*P*＜0.001）。尽管癌症中凝血功能的激活机制是多因素的，但是组织因子（tissue factor, TF）在这一过程中起重要作用。癌细胞TF表达水平的升高以及血小板、单核细胞和基质细胞释放TF，这都是促凝血活动的主要来源^[8]^。由这种跨膜糖蛋白启动的凝血级联反应触发了一系列事件，进而将凝血酶原转化为凝血酶，产生不溶性的纤维凝块。

本研究结果显示，NSCLC患者中鳞癌比腺癌的Fib水平升高（*P*＜0.001），与相关研究^[9]^结果一致。本研究表明，NSCLC患者不同TNM分期及淋巴结转移状态的APTT、Fib水平具有统计学差异（*P*＜0.001）。而研究^[10]^表明不同TNM分期和病理类型的肺癌患者的Fib和D-二聚体水平未见明显差异。现在许多研究对凝血功能指标如PT、APTT、D-二聚体和Plt等与肺癌患者病理分型、淋巴结是否转移和TNM分期等的关系进行了分析，但结论仍有所争议，需多中心大样本的研究进一步证实。

血液凝固是由许多凝血因子参与的、复杂的蛋白质酶解过程，包括内源性和外源性两条途径。APTT、PT是主要反映内、外源凝血系统各凝血因子的含量与活性的指标。内源途径是血液通过接触带负电荷的异物表面而启动，而外源途径通过TF触发其级联反应，其机制可能为TNF-α、IL-1β等细胞因子诱导血管内皮细胞、单核细胞等大量表达TF^[11]^，从而激活相关凝血因子形成复合物，从而使患者机体的血液系统转化为血栓前状态，继而形成血栓。研究^[12]^显示，PT的延长与NSCLC较差的预后密切相关，但是在这个研究中多变量模型没有证实凝血因子的预后相关性。本研究在单因素分析中也揭示了PT和INR延长的意义，在多因素生存分析中显示INR是肺癌的独立预后因素（*P*=0.017），与相关研究结果一致^[13]^。基于这些发现，在不远的将来PT、INR可能成为NSCLC预后指标。

纤溶系统的亢进也是高凝状态的重要的反应。Fib和D-D是反映体内存在纤溶亢进及高凝状态的较为特异性的指标。Fib由肝脏产生，是血浆中含量最高的凝血蛋白，被激活后转变为纤维蛋白多聚体，交联各种血细胞形成血凝块，保护循环中的癌细胞逃避免疫细胞的杀伤，从而增加肿瘤细胞转移的潜能，同时又可通过血小板膜表面糖蛋白介导激活血小板。D-D是交联的纤维蛋白裂解产生的一种代谢产物，其存在表明体内有纤维蛋白形成和溶解。

在一项肺癌转移研究中，学者们发现纤维蛋白原缺陷小鼠的肺部和局部淋巴结的转移率均低于野生型小鼠，而两种小鼠的血管生成及肿瘤的生长没有明显差异^[14, 15]^。而另有研究发现肿瘤细胞通过合成纤维蛋白原，并与成纤维细胞生长因子相互作用来促进肿瘤的生长^[16]^。我们研究发现NSCLC患者Fib和D-D水平升高（*P*＜0.001），表明机体存在纤溶亢进和高血凝状态，有利于血栓形成，这与相关的研究是一致的^[17, 18]^。肿瘤可激活血小板和血管内皮细胞产生纤溶抑制剂，从而抑制纤维蛋白的降解^[19]^。在本研究^[20]^中，高纤维蛋白原水平的患者生存率下降（*P*＜0.001）。这个结果与最近的一项研究相一致的，该研究报告了在结直肠癌中极高纤维蛋白原水平与较短的DFS之间的相关性。现在许多研究^[21-24]^已经证实，D-D的升高是NSCLC预后差的重要预测标志物。在本研究中D-D水平升高的患者有生存期下降的趋势，但是没有达到统计学意义（*P*=0.105）。

血小板是骨髓成熟的巨核细胞胞浆裂解脱落的活性胞质小块，具有粘附、聚集和释放的功能，直接参与血栓形成并在血栓形成过程中起关键性作用。研究^[25]^发现血小板增多存在于30%-60%的肿瘤患者中，肺癌患者血小板增多较普遍。我们研究发现NSCLC患者PLT水平较对照组升高（*P*=0.001, 5）；PLT水平高于中位数的患者其生存率有下降的趋势，但未达到统计学意义（P =0.156）。血小板通过释放其颗粒中的各种生长因子和趋化因子（如VEGF、PDGF、IGF-1等），而上调其它促血管新生介质的表达，如基质细胞衍生因子CXCL12、MMP-1、MMP-2和MMP-9等，增加血管生成，促进肿瘤的生长^[26, 27]^。肿瘤细胞所致的血小板聚集高度支持肿瘤转移^[28]^。PLT可通过蛋白酶类、白介素、凝血酶等介导产生，而激活的PLT有更强的粘附性和聚集性，提高血液凝固性，使机体处于高血凝状态，导致血栓性疾病的发生率增加。

肺癌患者常伴凝血及纤溶功能的异常，使机体内存在高凝状态，有利于血栓的形成。因此，对肺癌患者检测血浆中凝血及纤溶指标，早期发现血液高凝状态，及早采取适当的治疗措施，对避免VTE的发生及肺癌的预后可能有重要的临床意义。
